# Bidirectional Text Navigation (ColoPrep) Increases Colonoscopy Show Rate

**DOI:** 10.1016/j.gastha.2026.101006

**Published:** 2026-05-11

**Authors:** Samantha Boger, Nadim Mahmud, Catherine Reitz, Kristi Delp, Nuzhat Ahmad, Shivan J. Mehta

**Affiliations:** 1 Division of Gastroenterology & Hepatology, Perelman School of Medicine, University of Pennsylvania, Philadelphia, Pennsylvania; 2 Population Health Lab, Center for Health Care Transformation & Innovation, University of Pennsylvania, Philadelphia, Pennsylvania

Colonoscopy is important for reducing colorectal cancer (CRC) burden, but it requires preparation including altering diet, taking a high volume of laxatives, and potentially adjusting medications. The complexity of preparation leads to substantial cancellation and no-show rates, along with suboptimal bowel preparation. Phone calls by clinicians and staff can increase adherence, but this high-resource intervention is challenging to scale.^1^^,^^2^ At our health system, a pilot bidirectional text intervention showed promise at improving show rate, but a subsequent trial with unidirectional messaging did not show improvement.^3^^,^^4^

This study evaluated the effect of the ColoPrep bidirectional text navigation program by comparing show rate and preparation quality before and after implementation. Data were analyzed across 3 outpatient endoscopy suites at an academic health center in the Philadelphia region using a difference-in-differences design. All 3 clinical practices included faculty physicians with advanced practice providers and fellows, and both direct access patients scheduled through a centralized call center and previously seen gastroenterology patients. ColoPrep was implemented at Hospital 1 and 2, leaving Hospital 3 to serve as a natural control. This project was reviewed and determined to qualify as quality improvement by the University of Pennsylvania’s Institutional Review Board.

ColoPrep (Way to Health) provides timely reminders to patients, answers common questions, and connects patients to staff for more in-depth inquiries (Supplementary Table).^5^ A Health Level 7 data feed enrolls patients automatically at the time of scheduling in the OpTime (Epic) surgical management tool. All outpatient colonoscopy and dual colonoscopy/endoscopy procedures are included. Text messaging begins the day the appointment is scheduled, and patients are given the opportunity to opt-out at any time. Messages related to appointment logistics (cancels, reschedules, appointment date/time, and transportation) are managed by administrative staff. Cancellations are confirmed via text, while reschedules must be managed by phone. Messages related to the preparation (medications, liquid diet, clear liquids, timing of the preparation, low fiber diet) are answered by administrative staff after consultation with clinical staff. If more nuanced questions arise, clinical staff will reach out by phone to discuss with the patient.

Data on colonoscopy appointment completion (including sigmoidoscopy) were collected for the preintervention period (October 12, 2022–March 31, 2023) and the postintervention period (October 12, 2023–March 31, 2024). April 2023 to September 2023 served as a wash out period, since ColoPrep could only be implemented when the appointments were scheduled. Race and ethnicity are included according to a self-report in the electronic health record. Using intention-to-treat principles, all patients in the intervention group were included, even if they did not engage with ColoPrep.

The primary outcome was the proportion of colonoscopies completed out of those that were scheduled (completion rate), including all reasons for incomplete procedures (patient cancellation, rescheduling, no-show, physician cancellation, aborted procedures due to poor bowel preparation or medical complication). The secondary outcome of physician-reported bowel preparation quality was dichotomized as follows: excellent, good, and adequate were categorized as “adequate,” while fair, obscured, unsatisfactory, inadequate, poor, and not reported as “inadequate.”

Completion rates were collapsed by facility, month, and time period. The change in completion rate was calculated by subtracting the postintervention period completion rate by the preintervention rate. Unadjusted linear regression was used to compare the changes between intervention and control facilities. Secondary analysis was conducted including only Black patients, since there are known racial disparities in colorectal cancer screening and outcomes. Using a similar analytical approach, we also analyzed the proportion of completed colonoscopies with adequate bowel preparation. The analysis was completed from June 1 to November 1, 2024.

The study included 18,929 patients in the intervention group and 17,231 in the control group; 55.3% female, 55.6% White, 34.1% Black, and 34.8% had a gastroenterology visit in the prior year (Table). Primary analysis showed a 5.9 percentage point greater increase in colonoscopy completion rate at hospitals which implemented ColoPrep (β = 0.061; *P* < .013; confidence interval [CI] 0.015–0.106) from the preintervention to postintervention time frame (Supplementary Figure). When sigmoidoscopy was excluded from analysis, there was an 8.4 percentage point greater increase in colonoscopy completion rate at intervention sites from preintervention to postintervention period (β = 0.085; *P* < .001; CI 0.046–0.124). Secondary analysis of Black patients (7541 intervention, 5872 control) showed similar results to the primary analysis, with a 6.3 percentage point greater increase in colonoscopy completion rate at intervention sites (β = 0.068; *P* < .067; CI −0.0058 to 0.14). When bowel preparation quality was analyzed, the percentage of patients with adequate bowel preparation decreased by 0.54 percentage points at the control site and increased by 0.25 percentage points at intervention sites after intervention. There was no statistically significant change in bowel prep quality (β = −0.006; *P* < .568; CI [−0.031 to 0.018]).

**Table:** Participant Characteristics

Characteristics	Hospital 1 (intervention) (N = 10,611)	Hospital 2 (intervention) (N = 8,315)	Hospital 3 (control) (N = 17,231)
Age, mean (SD)	56.9 (13.4)	57.6 (13.4)	57.6 (13.7)
Time period			
Preintervention	5640 (53.2%)	3882 (46.7%)	8534 (49.5%)
Postintervention	4971 (46.8%)	4433 (53.3%)	8697 (50.5%)
Sex			
Female	5841 (55.0%)	4839 (58.2%)	9524 (55.3%)
Male	4766 (44.9%)	3476 (41.8%)	7704 (44.7%)
Other/unknown	4 (<1%)	0 (0.0%)	3 (<1%)
Insurance type			
Medicaid	1753 (16.6%)	927 (11.1%)	2113 (12.3%)
Medicare	3345 (31.5%)	2683 (32.2%)	5809 (33.7%)
Patient declined	209 (2.0%)	111 (1.3%)	259 (1.5%)
Private	5285 (49.8%)	4590 (55.2%)	9027 (52.4%)
Self-pay	14 (0.1%)	4 (<1%)	23 (0.1%)
Race			
American Indian/Alaskan Native	24 (0.2%)	24 (0.3%)	34 (0.2%)
Asian	431 (4.1%)	387 (4.7%)	724 (4.2%)
Black	5181 (48.8%)	2360 (28.4%)	5872 (34.1%)
Native Hawaiian/Pacific Islander	11 (0.1%)	20 (0.2%)	18 (0.1%)
Other	697 (6.6%)	533 (6.4%)	999 (5.8%)
White	4267 (40.2%)	4991 (60.0%)	9584 (55.6%)
Ethnicity			
Hispanic Latino	350 (3.3%)	320 (3.8%)	608 (3.5%)
Not Hispanic or Latino	10,135 (95.5%)	7874 (94.7%)	16,436 (95.4%)
Patient declined	126 (1.2%)	121 (1.5%)	187 (1.1%)
GI visit in past year			
No	7988 (75.3%)	6027 (72.5%)	11,231 (65.2%)
Yes	2623 (24.7%)	2288 (27.5%)	6000 (34.8%)
Appointment status (including both time periods)			
Canceled	4066 (38.3%)	2561 (30.8%)	7515 (43.6%)
Completed	6545 (61.7%)	5754 (69.2%)	9716 (56.4%)
Reason for cancel			
Medical	160 (3.9%)	102 (4.0%)	152 (2.0%)
Not described	4 (0.1%)	16 (0.6%)	84 (1.1%)
Other	427 (10.5%)	903 (35.3%)	1290 (17.2%)
Patient	2702 (66.5%)	1444 (56.4%)	4911 (65.3%)
Provider	773 (19.0%)	96 (3.7%)	1078 (14.3%)

GI, gastroenterology; SD, standard deviation.

In this study, we found that patients enrolled in bidirectional text navigation had significantly higher colonoscopy show rates as compared to those receiving usual care. Similar results were seen in subgroup analysis including only Black patients, indicating that this technology-focused intervention did not exacerbate racial disparities. There was no impact seen in bowel preparation score of the colonoscopies completed, although potential improvement is probably reflected in the completion rate.

We posit 2 primary reasons for the improved show rates. First, it provided real-time nudges to patients in a user-friendly format, helping patients overcome information complexity and forgetfulness. Second, bidirectional texting added a level of responsibility and engagement with the program, while allowing patients to ask questions and easily communicate with providers. Similar results have been seen in other randomized studies, and digital navigation is a weak recommendation by the US Multi-Society Task Force on Colorectal Cancer.^6^, ^7^, ^8^, ^9^

The main strength of the study was its quasi-experimentation design to both compare the change over time and among the intervention and control arm, demonstrating how text navigation functions in a real-world setting with a diverse patient population. The main limitation was that there may have been other initiatives at individual sites that could be contributing to the completion rate, and there was a lower show rate in the control hospital during the preintervention period.

Our results suggest that automated text messaging programs can improve colonoscopy attendance, enhancing efficiency and outcomes for endoscopy centers. Future studies can evaluate the use of artificial intelligence to personalize communications.

## Authors’ Contributions

Samantha Boger contributed to the study concept and design, acquisition of data, analysis and interpretation of data, drafting of the manuscript, and statistical analysis. Nadim Mahmud contributed to the study concept and design, analysis and interpretation of the data, statistical analysis, and critical revision of the manuscript for important intellectual content. Catherine Reitz contributed to the study concept and design, acquisition of the data, analysis and interpretation of the data, and critical revision of the manuscript for important intellectual content. Kristi Delp contributed to the analysis and interpretation of the data and critical revision of the manuscript for important intellectual content. Nuzhat Ahmad contributed to the analysis and interpretation of the data and critical revision of the manuscript for important intellectual content. Shivan J. Mehta contributed to the study concept and design, analysis and interpretation of data, statistical analysis, critical revision of the manuscript for important intellectual content, obtained funding, and study supervision.

## Supplementary Materials

Supplementary Table and Supplementary Figure

Extended PDF
