# Supplementary material for: Bidirectional Text Navigation (ColoPrep) Increases Colonoscopy Show Rate
Source: Gastro Hep Adv. 2026 May 11;5(8):101006. doi: 10.1016/j.gastha.2026.101006 (PMC13262132; doi:10.1016/j.gastha.2026.101006)

Supplemental Materials

Supplement Table 1: Text messaging content for ColoPrep intervention…………………………...2

Supplement Figure 1: Pre and Post-Intervention Period Completion Rate for Control and Intervention………………………………………………………………………………………..6

Supplement Table 1. Text messaging content for ColoPrep intervention

Time	Message
Appt Scheduled	Hi {PARTICIPANT_FIRSTNAME}, this is Penn Medicine. Congrats on scheduling your colonoscopy for {{#appointment_date}} @ time with Dr.{{#provider_last_name}}! Penn GI would like to offer you a texting program to guide you through the prep process in the week prior to your colonoscopy. Just so you have it, here's an link to the prep instructions: https://w2h.us/prepguide<br><br>Please be advised that msg & data rates may apply and standard SMS texting is not secure, so other people may be able to see the information in the messages. Text BYE if you do not wish to receive text messages from this program.
30 & 13 Days Prior to Appointment Date	Hi PARTICIPANT_FIRSTNAME! This is Penn Medicine with a reminder that your colonoscopy is coming up on {{@appointment_date}} at {{@appointment_time}} (with an arrival time of {{@arrival_time}}). Reply "1" to confirm, "2" to reschedule, "3" to cancel.<br><br>1 – Confirm<br><br>2 – Reschedule<br><br>3 – Cancel<br><br>Reply if Confirm:<br><br>Great! We'll be in touch with more info a week before your appointment. As a reminder, you'll need a responsible adult to take you home safely from your procedure. Here's a link to your instructions for the prep process: https://w2h.us/prepguide<br><br>Reply if Reschedule:<br><br>Ok, thank you for letting us know you'd like to reschedule. We are not able to reschedule via text. Please call us at {{@clinic_number}} for assistance with rescheduling. Your appointment will NOT be changed unless you speak with someone or reply CANCEL to this message. (In-basket msg sent to Coordinator)<br><br>Reply if Cancel:<br><br>Ok thank you for letting us know you'd like to cancel. If you cancel now it may not be possible to reschedule you right away. Reply 1 if you are SURE you want to cancel. Your appointment will NOT be canceled if you do not reply to this message. If you have any other questions about your appointment, please reply SUPPORT instead.<br><br>1 – I'm Sure I Want to Cancel<br><br>Reply if 1:<br><br>Ok thank you. Your appointment will be cancelled within 1 business day. Until it is canceled, you may continue to receive messages from this program. (In-basket msg sent to Coordinator)<br><br>If No Reply:<br><br>The next day at 8am: We didn't hear back from you so we will plan to see you on {{@appointment_date}} for your procedure. If you need to cancel, please reply CANCEL to this message. (In-basket msg sent to Coordinator)

Day 14	Make sure you arrange with someone to take you home after your procedure. Have you made arrangements with someone? Reply Y or N.<br><br>If Y: Great! Let your ride know that the exam should finish 2-3 hours after your arrival time. Your recovery nurse will call them when you're ready to leave, and your ride should pick you up within 30 minutes of that call.<br><br>If N: Ok, but please do that soon! It takes time for the anesthesia to wear off, so you need a responsible adult to take you home safely. Your person may take you home using public transportation (SEPTA), a ride-share like Uber or Lyft, or a taxi, but you may not go home alone. If you don't have someone to take you home, we will not be able to complete your procedure. (In-basket msg sent to Coordinator)<br><br>If No Reply: The next day at 8am: We didn't hear back from you. Please be sure to have a responsible person to bring you home approximately 2-3 hours after your arrival time. If you do not have someone to take you home, we will not be able to complete your<br><br>procedure. Text SUPPORT any time for additional information. (In-basket msg sent to Coordinator)
Day 8, 8am	This program has been created to help patients complete a standard, one-day colonoscopy preparation using over-the-counter preparation supplies (Miralax, Gatorade, etc). Most patients complete their preparation in this way, starting the day before their procedure. However, if your Dr. has prescribed you a different preparation (a two-day (extended) prepration, or SuPrep, SuTab, Moviprep, or GoLytly preparation) , you should follow those preparation instructions instead of those provided by this program. Which preparation were you prescribed?<br><br>1) Standard over-the-counter (Miralax, Gatorade, etc)<br><br>2) A two-day extended preparation<br><br>3) Suprep, SuTab, Moviprep, or GoLytly (or other Rx that must be filled at a pharmacy)<br><br>4) I'm not sure – help?<br><br>If 1: Great, your daily preparation text message guide will begin tomorrow. You may read the instructions in full or watch an instructional video here: https://w2h.us/prepguide<br><br>If 2: Ok, we're sorry that this is not the program for you. Please follow the preparation instructions that were provided to you by your provider. Your messaging will now be turned off and this program will no longer be active. If you have received this message in error, reply with the word PREP to resume your preparation program. Thank you! (W2H Action: messaging automatically turns OFF)<br><br>If 3: Ok, we're sorry that this is not the program for you. You may read the instructions for your preparation process here: https://w2h.us/PEGPrep Your messaging will now be turned off and this program will no longer be active. If you have received this message in error, reply with the word PREP to resume your preparation program. Thank you! (W2H Action: messaging automatically turns OFF)<br><br>If 4: We will notify a staff member to help clarify which preparation process you should follow. Someone will reach out to you as soon as possible, but if you need to reach someone more quickly, please call {{@clinic_number}}. (W2H Action: In-basket msg sent to Coordinator)<br><br>If PREP: Your colonoscopy preparation program (using the Miralax/Gatorade preparation) will now resume. If you have questions or need assistance, reply with the word SUPPORT. (W2H Action: messaging automatically turns ON)

Day 7, 8am	It’s officially prep week! We hope this program will help you through your prep! Please review this list of medications carefully: https://w2h.us/prepguide If you are on any of those listed, any blood thinners (like Coumadin, Warfarin, Eliquis, Xarelto, Pradaxa), or any other medication for diabetes, ask your prescribing doctor if you should adjust your medications before this procedure. Please do this as soon as possible. If you are taking an iron supplement, please stop this immediately until after your procedure. Other medications should be taken as instructed.
Day 6, 8am	Good morning, PARTICIPANT_FIRSTNAME! Don’t forget to purchase your prep supplies from the local pharmacy or grocery store! Your prep includes:<br>- MiraLAX: 1 bottle, 238g<br>- Dulcolax: 4 pills, 5 mgs each<br>- Clear electrolyte drink: 64 ounces<br>Clear electrolyte drinks include Gatorade, PowerAde, Propel Fitness Water, or Pedialyte (yellow, green, or clear flavors ONLY). Please use the zero sugar versions if you are diabetic.
Day 5, 8am	Good morning, <name>! Let’s take a look at your diet in preparation for your colonoscopy. Fiber is hard to purge from your system while doing a bowel prep. Starting today, please limit high fiber foods like fruits, veggies, nuts and seeds, and focus on low-fiber foods such as yogurt, eggs, white bread, rice, chicken, pork, and fish<br>It is also extremely beneficial to start drinking more water. The better hydrated you are, the more success you will have with your prep. Remember you can text “Support” at any time for additional information.
Day 3, 8am	Hi PARTICIPANT_FIRSTNAME! Remember to avoid high-fiber foods: fruits, vegetables, nuts, and seeds<br>Eat Low-Fiber foods: yogurt, egg, white bread, rice, chicken, pork, and fish. Do you have any questions we can answer for you today? Reply Y or N.<br>If N: Great, you’re a pro! Text “Support” at any time if you change your mind.<br>If Y: Ok, in a few words, please tell us what you need additional assistance with and a staff member will get back to you within one business day. If you need to speak with someone more quickly, please call: {{@clinic_number}} for assistance. If this is
Day 2, 2pm	Starting tomorrow morning, you should only have clear liquids until your procedure is complete. Reminder: have all of your prep supplies on hand. For complete instructions, click here:<br>https://w2h.us/prepguide [attachment]:<br>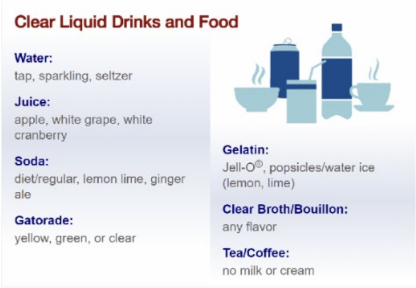
Day 2, 5pm	Please enjoy your dinner (with low fiber foods)! This will be your last meal with solid food before

	your colonoscopy. Remember, as soon as you wake up tomorrow, consume ONLY clear liquids until your procedure is complete.
Day 1, 8am	Good morning PARTICIPANT_FIRSTNAME! It's game time. You may only consume clear liquids from now until your procedure is complete. You can do this! More instructions to follow this afternoon when it's time to start your prep.
Day 1, 4pm	Time to get started.! Take the 4 Dulcolax pills now. Then, at 5 PM, mix the entire bottle of MiraLAX with the 64 oz of an electrolyte drink (Gatorade or Powerade, etc) and drink the first half of this mixture (32 oz). If you feel nauseous, you can always slow down to help tolerate it. Try drinking about 8 ounces every 15 minutes. We'll let you know when to drink the other half. You can do it!
Day 1, 8pm	Just to confirm, your arrival time is {{@arrival_time}} tomorrow. Great job, the first half of your prep is complete! Start drinking the other half of the MiraLAX solution 6 hours before your arrival (at {{@prep_time}}), and stop drinking all clear liquids 3 hours before at (at {{@stopliquids_time}}). If you have any questions overnight, please text Support. You're in the final stretch
Day 0, 5am or 3 hours before arrival time	You've come so far and the worst is over! Wear comfortable clothes today, and leave your valuables (like credit cards, cell phone, cash and jewelry) at home or with your ride. Please be SURE to bring your cell phone. Your appointment is at {{@clinic_address}}. Please arrive by {{@arrival_time}}.<br><br>When you arrive, please [parking and building instructions]. Your ride may come in with you. Congratulations! You did it
Support- help on-demand	A good prep is key to your procedure! We'll do our best to answer your question, please reply with the number (1-6) of the area you need help:<br><br>1: Appointment Info<br><br>2: Ride<br><br>3. Diet<br><br>4. Prep supplies and instructions<br><br>5. Prescriptions<br><br>6. Something else<br><br>After auto reply for any topic listed:<br><br>Does this answer your question? Reply Y or N<br><br>If Y: Great – we're happy we could help! You can text "Support" again at any time if you have any additional questions.<br><br>If N or Option 6: We're sorry our automated support couldn't help you with that. We will have someone reach out within the next business day. If you need to reach someone more quickly, please call the {{@clinic_name}} office directly at {{@clinic_number}}. (In-basket msg sent to Coordinator)

Supplement Figure 1. Pre and Post-Intervention Period Completion Rate for Control and Intervention

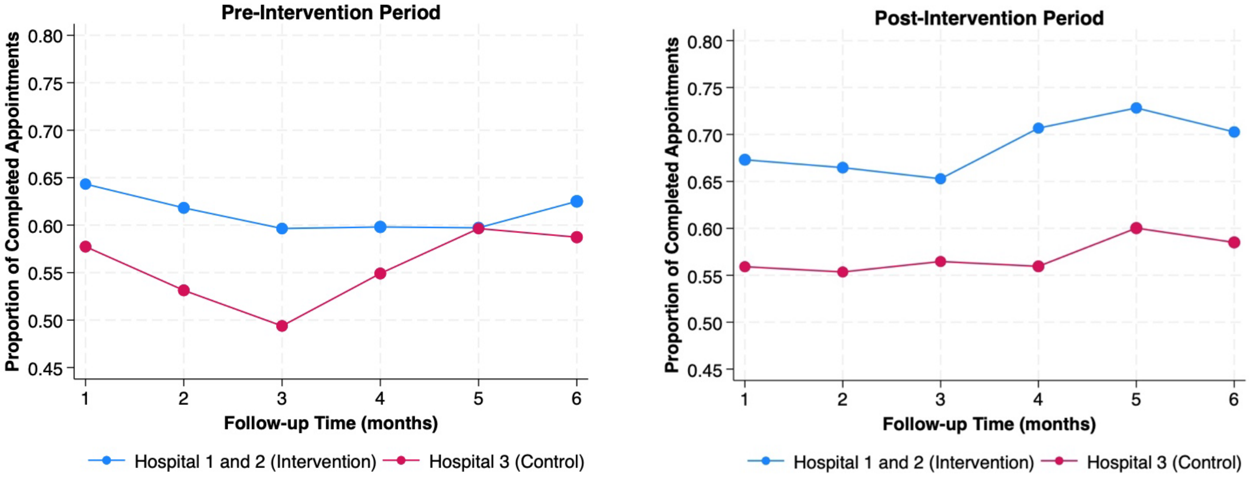

Supplement: Supplementary Table and Supplementary Figure [file mmc1.pdf]
