# Supplementary material for: Bidirectional Text Navigation (ColoPrep) Increases Colonoscopy Show Rate
Source: Gastro Hep Adv. 2026 May 11;5(8):101006. doi: 10.1016/j.gastha.2026.101006 (PMC13262132; doi:10.1016/j.gastha.2026.101006)

Gastro Hep Advances 2026;5:101006

RESEARCH LETTER

Bidirectional Text Navigation (ColoPrep) Increases Colonoscopy Show Rate

Colonoscopy is important for reducing colorectal cancer (CRC) burden, but it requires preparation including altering diet, taking a high volume of laxatives, and potentially adjusting medications. The complexity of preparation leads to substantial cancellation and no-show rates, along with suboptimal bowel preparation. Phone calls by clinicians and staff can increase adherence, but this high-resource intervention is challenging to scale.[1,2] At our health system, a pilot bidirectional text intervention showed promise at improving show rate, but a subsequent trial with unidirectional messaging did not show improvement.[3,4]

This study evaluated the effect of the ColoPrep bidirectional text navigation program by comparing show rate and preparation quality before and after implementation. Data were analyzed across 3 outpatient endoscopy suites at an academic health center in the Philadelphia region using a difference-in-differences design. All 3 clinical practices included faculty physicians with advanced practice providers and fellows, and both direct access patients scheduled through a centralized call center and previously seen gastroenterology patients. ColoPrep was implemented at Hospital 1 and 2, leaving Hospital 3 to serve as a natural control. This project was reviewed and determined to qualify as quality improvement by the University of Pennsylvania's Institutional Review Board.

ColoPrep (Way to Health) provides timely reminders to patients, answers common questions, and connects patients to staff for more in-depth inquiries (Supplementary Table).[5] A Health Level 7 data feed enrolls patients automatically at the time of scheduling in the OpTime (Epic) surgical management tool. All outpatient colonoscopy and dual colonoscopy/endoscopy procedures are included. Text messaging begins the day the appointment is scheduled, and patients are given the opportunity to opt-out at any time. Messages related to appointment logistics (cancels, reschedules, appointment date/time, and transportation) are managed by administrative staff. Cancellations are confirmed via text, while reschedules must be managed by phone. Messages related to the preparation (medications, liquid diet, clear liquids, timing of the preparation, low fiber diet) are answered by administrative staff after consultation with clinical staff. If more nuanced questions arise, clinical staff will reach out by phone to discuss with the patient.

Data on colonoscopy appointment completion (including sigmoidoscopy) were collected for the preintervention period (October 12, 2022–March 31, 2023) and the postintervention period (October 12, 2023–March 31, 2024). April 2023 to September 2023 served as a wash out period, since ColoPrep could only be implemented when the appointments were scheduled. Race and ethnicity are included according to a self-report in the electronic health record. Using intention-to-treat principles, all patients in the intervention group were included, even if they did not engage with ColoPrep.

The primary outcome was the proportion of colonoscopies completed out of those that were scheduled (completion rate), including all reasons for incomplete procedures (patient cancellation, rescheduling, no-show, physician cancellation, aborted procedures due to poor bowel preparation or medical complication). The secondary outcome of physician-reported bowel preparation quality was dichotomized as follows: excellent, good, and adequate were categorized as "adequate," while fair, obscured, unsatisfactory, inadequate, poor, and not reported as "inadequate."

Completion rates were collapsed by facility, month, and time period. The change in completion rate was calculated by subtracting the post-intervention period completion rate by the preintervention rate. Unadjusted linear regression was used to compare the changes between intervention and control facilities. Secondary analysis was conducted including only Black patients, since there are known racial disparities in colorectal cancer screening and outcomes. Using a similar analytical approach, we also analyzed the proportion of completed colonoscopies with adequate bowel preparation. The analysis was completed from June 1 to November 1, 2024.

The study included 18,929 patients in the intervention group and 17,231 in the control group; 55.3% female, 55.6% White, 34.1% Black, and 34.8% had a gastroenterology visit in the prior year (Table). Primary analysis showed a 5.9 percentage point greater increase in colonoscopy completion rate at hospitals which implemented ColoPrep ($\beta = 0.061$; $P < .013$; confidence interval [CI] 0.015–0.106) from the preintervention to post-intervention time frame (Supplementary Figure). When sigmoidoscopy was excluded from analysis, there was an 8.4 percentage point greater increase in colonoscopy completion rate at intervention sites from preintervention to post-intervention period ($\beta = 0.085$; $P < .001$; CI 0.046–0.124). Secondary analysis of Black patients (7541 intervention, 5872 control) showed similar results to the primary analysis, with a 6.3 percentage point greater increase in colonoscopy completion rate at intervention sites ($\beta = 0.068$; $P < .067$; CI -0.0058 to 0.14). When bowel preparation quality was analyzed, the percentage of patients with adequate bowel preparation decreased by 0.54 percentage points at the control site and increased by 0.25 percentage points at intervention sites after intervention. There was no

Table. Participant Characteristics

Characteristics	Hospital 1 (intervention) (N = 10,611)	Hospital 2 (intervention) (N = 8,315)	Hospital 3 (control) (N = 17,231)
Age, mean (SD)	56.9 (13.4)	57.6 (13.4)	57.6 (13.7)
Time period			
Preintervention	5640 (53.2%)	3882 (46.7%)	8534 (49.5%)
Postintervention	4971 (46.8%)	4433 (53.3%)	8697 (50.5%)
Sex			
Female	5841 (55.0%)	4839 (58.2%)	9524 (55.3%)
Male	4766 (44.9%)	3476 (41.8%)	7704 (44.7%)
Other/unknown	4 (<1%)	0 (0.0%)	3 (<1%)
Insurance type			
Medicaid	1753 (16.6%)	927 (11.1%)	2113 (12.3%)
Medicare	3345 (31.5%)	2683 (32.2%)	5809 (33.7%)
Patient declined	209 (2.0%)	111 (1.3%)	259 (1.5%)
Private	5285 (49.8%)	4590 (55.2%)	9027 (52.4%)
Self-pay	14 (0.1%)	4 (<1%)	23 (0.1%)
Race			
American Indian/Alaskan Native	24 (0.2%)	24 (0.3%)	34 (0.2%)
Asian	431 (4.1%)	387 (4.7%)	724 (4.2%)
Black	5181 (48.8%)	2360 (28.4%)	5872 (34.1%)
Native Hawaiian/Pacific Islander	11 (0.1%)	20 (0.2%)	18 (0.1%)
Other	697 (6.6%)	533 (6.4%)	999 (5.8%)
White	4267 (40.2%)	4991 (60.0%)	9584 (55.6%)
Ethnicity			
Hispanic Latino	350 (3.3%)	320 (3.8%)	608 (3.5%)
Not Hispanic or Latino	10,135 (95.5%)	7874 (94.7%)	16,436 (95.4%)
Patient declined	126 (1.2%)	121 (1.5%)	187 (1.1%)
GI visit in past year			
No	7988 (75.3%)	6027 (72.5%)	11,231 (65.2%)
Yes	2623 (24.7%)	2288 (27.5%)	6000 (34.8%)
Appointment status (including both time periods)			
Canceled	4066 (38.3%)	2561 (30.8%)	7515 (43.6%)
Completed	6545 (61.7%)	5754 (69.2%)	9716 (56.4%)
Reason for cancel			
Medical	160 (3.9%)	102 (4.0%)	152 (2.0%)
Not described	4 (0.1%)	16 (0.6%)	84 (1.1%)
Other	427 (10.5%)	903 (35.3%)	1290 (17.2%)
Patient	2702 (66.5%)	1444 (56.4%)	4911 (65.3%)
Provider	773 (19.0%)	96 (3.7%)	1078 (14.3%)

GI, gastroenterology; SD, standard deviation.

statistically significant change in bowel prep quality ($\beta = -0.006$; $P < .568$; CI [−0.031 to 0.018]).

In this study, we found that patients enrolled in bidirectional text navigation had significantly higher colonoscopy show rates as compared to those receiving usual care. Similar results were seen in subgroup analysis including only Black patients, indicating that this technology-focused intervention did not exacerbate racial disparities. There was no impact seen in bowel preparation score of the colonoscopies completed, although potential improvement is probably reflected in the completion rate.

We posit 2 primary reasons for the improved show rates. First, it provided real-time nudges to patients in a user-friendly format, helping patients overcome information complexity and forgetfulness. Second, bidirectional texting added a level of responsibility and engagement with the program, while allowing patients to ask questions and easily communicate with providers. Similar results have been seen in other randomized studies, and digital navigation is a weak recommendation by the US Multi-Society Task Force on Colorectal Cancer.[6–9]

The main strength of the study was its quasi-experimentation design to both compare the change over time and among the intervention and control arm, demonstrating how text navigation functions in a real-world setting with a diverse patient population. The main limitation was that there may have been other initiatives at individual sites that could be contributing to the completion rate, and there was a lower show rate in the control hospital during the pre-intervention period.

Our results suggest that automated text messaging programs can improve colonoscopy attendance, enhancing efficiency and outcomes for endoscopy centers. Future studies can evaluate

the use of artificial intelligence to personalize communications.

Authors' Contributions

Samantha Boger contributed to the study concept and design, acquisition of data, analysis and interpretation of data, drafting of the manuscript, and statistical analysis. Nadim Mahmud contributed to the study concept and design, analysis and interpretation of the data, statistical analysis, and critical revision of the manuscript for important intellectual content. Catherine Reitz contributed to the study concept and design, acquisition of the data, analysis and interpretation of the data, and critical revision of the manuscript for important intellectual content. Kristi Delp contributed to the analysis and interpretation of the data and critical revision of the manuscript for important intellectual content. Nuzhat Ahmad contributed to the analysis and interpretation of the data and critical revision of the manuscript for important intellectual content. Shivan J. Mehta contributed to the study concept and design, analysis and interpretation of data, statistical analysis, critical revision of the manuscript for important intellectual content, obtained funding, and study supervision.

SAMANTHA BOGER[1,2]
NADIM MAHMUD[1]
CATHERINE REITZ[1,2]
KRISTI DELP[1]
NUZHAT AHMAD[1]
SHIVAN J. MEHTA[1,2]
[1]Division of Gastroenterology & Hepatology, Perelman School of Medicine, University of Pennsylvania, Philadelphia, Pennsylvania
[2]Population Health Lab, Center for Health Care Transformation & Innovation, University of Pennsylvania, Philadelphia, Pennsylvania

Correspondence:
Address correspondence to: Shivan J. Mehta, MD, MBA, MSHP, Perelman School of Medicine, University of Pennsylvania, 3600 Civic Center Blvd, 6W-201a, Philadelphia, Pennsylvania 19104. e-mail: shivan.mehta@pennmedicine.upenn.edu.

Supplementary Materials

Material associated with this article can be found, in the online version, at https://doi.org/10.1016/j.gastha.2026.101006.

References

1. DeGroff A, et al. Am J Prev Med 2017;53(3):363–372.
2. Liu X, et al. Gut 2014; 63(1):125–130.
3. Mahmud N, et al. Health Educ Behav 2019;46(6):942–946.
4. Mahmud N, et al. JAMA Netw Open 2021;4(1):e2034553-e.
5. Asch DA, et al. LDI Issue Brief 2012;17(9):1–4.
6. Solonowicz O, et al. J Clin Gastroenterol 2022;56(2):166–172.
7. Desai M, et al. Endosc Int Open 2019;07(02):E216–E224.
8. Walter B, et al. Gastrointest Endosc 2019;89(3):506–513.e4.
9. Jacobson BC, et al. Gastroenterology 2025;168(4):798–829.

Abbreviations used in this paper: **CRC, colorectal cancer; CI, confidence interval**

Most current article

© 2026 The Author(s). Published by Elsevier Inc. on behalf of American Gastroenterological Association Institute. This is an open access article under the CC BY license (http://creativecommons.org/licenses/by/4.0/).
2772-5723
https://doi.org/10.1016/j.gastha.2026.101006

Received December 19, 2025. Accepted May 5, 2026.

Conflicts of Interest:
The author discloses the following: Shivan J. Mehta has received compensation from Guardant Health. The remaining authors disclose no conflicts.

Funding:
This trial was supported by grant number R25 DK108711-01 from the National Institute of Diabetes and Digestive and Kidney Diseases of the National Institutes of Health and the Marc J. Leder Cancer Innovation Fund.

Ethical Statement:
This project was reviewed and determined to qualify as quality improvement by the University of Pennsylvania's Institutional Review Board.

Data Transparency Statement:
Outreach content available in the Supplement. Otherwise, patient data will not be shared.

Reporting Guidelines:
SQUIRE 2.0.

Supplemental information

Bidirectional Text Navigation (ColoPrep) Increases Colonoscopy Show Rate

Samantha Boger, Nadim Mahmud, Catherine Reitz, Kristi Delp, Nuzhat Ahmad, and Shivan J. Mehta

Supplemental Materials

Supplement Table 1: Text messaging content for ColoPrep intervention…………………………...2

Supplement Figure 1: Pre and Post-Intervention Period Completion Rate for Control and Intervention………………………………………………………………………………………..6

Supplement Table 1. Text messaging content for ColoPrep intervention

**Time**	**Message**
Appt Scheduled	Hi {PARTICIPANT_FIRSTNAME}, this is Penn Medicine. Congrats on scheduling your colonoscopy for {{#appointment_date}} @ time with Dr.{{#provider_last_name}}! Penn GI would like to offer you a texting program to guide you through the prep process in the week prior to your colonoscopy. Just so you have it, here's an link to the prep instructions: https://w2h.us/prepguide<br><br>Please be advised that msg & data rates may apply and standard SMS texting is not secure, so other people may be able to see the information in the messages. Text BYE if you do not wish to receive text messages from this program.
30 & 13 Days Prior to Appointment Date	Hi PARTICIPANT_FIRSTNAME! This is Penn Medicine with a reminder that your colonoscopy is coming up on {{@appointment_date}} at {{@appointment_time}} (with an arrival time of {{@arrival_time}}). Reply "1" to confirm, "2" to reschedule, "3" to cancel.<br><br>1 – Confirm<br><br>2 – Reschedule<br><br>3 – Cancel<br><br>Reply if Confirm:<br><br>Great! We'll be in touch with more info a week before your appointment. As a reminder, you'll need a responsible adult to take you home safely from your procedure. Here's a link to your instructions for the prep process: https://w2h.us/prepguide<br><br>Reply if Reschedule:<br><br>Ok, thank you for letting us know you'd like to reschedule. We are not able to reschedule via text. Please call us at {{@clinic_number}} for assistance with rescheduling. Your appointment will NOT be changed unless you speak with someone or reply CANCEL to this message. (In-basket msg sent to Coordinator)<br><br>Reply if Cancel:<br><br>Ok thank you for letting us know you'd like to cancel. If you cancel now it may not be possible to reschedule you right away. Reply 1 if you are SURE you want to cancel. Your appointment will NOT be canceled if you do not reply to this message. If you have any other questions about your appointment, please reply SUPPORT instead.<br><br>1 – I'm Sure I Want to Cancel<br><br>Reply if 1:<br><br>Ok thank you. Your appointment will be cancelled within 1 business day. Until it is canceled, you may continue to receive messages from this program. (In-basket msg sent to Coordinator)<br><br>If No Reply:<br><br>The next day at 8am: We didn't hear back from you so we will plan to see you on {{@appointment_date}} for your procedure. If you need to cancel, please reply CANCEL to this message. (In-basket msg sent to Coordinator)

Day 14	Make sure you arrange with someone to take you home after your procedure. Have you made arrangements with someone? Reply Y or N.<br><br>If Y: Great! Let your ride know that the exam should finish 2-3 hours after your arrival time. Your recovery nurse will call them when you're ready to leave, and your ride should pick you up within 30 minutes of that call.<br><br>If N: Ok, but please do that soon! It takes time for the anesthesia to wear off, so you need a responsible adult to take you home safely. Your person may take you home using public transportation (SEPTA), a ride-share like Uber or Lyft, or a taxi, but you may not go home alone. If you don't have someone to take you home, we will not be able to complete your procedure. (In-basket msg sent to Coordinator)<br><br>If No Reply: The next day at 8am: We didn't hear back from you. Please be sure to have a responsible person to bring you home approximately 2-3 hours after your arrival time. If you do not have someone to take you home, we will not be able to complete your<br><br>procedure. Text SUPPORT any time for additional information. (In-basket msg sent to Coordinator)
Day 8, 8am	This program has been created to help patients complete a standard, one-day colonoscopy preparation using over-the-counter preparation supplies (Miralax, Gatorade, etc). Most patients complete their preparation in this way, starting the day before their procedure. However, if your Dr. has prescribed you a different preparation (a two-day (extended) prepration, or SuPrep, SuTab, Moviprep, or GoLytly preparation) , you should follow those preparation instructions instead of those provided by this program. Which preparation were you prescribed?<br><br>1) Standard over-the-counter (Miralax, Gatorade, etc)<br><br>2) A two-day extended preparation<br><br>3) Suprep, SuTab, Moviprep, or GoLytly (or other Rx that must be filled at a pharmacy)<br><br>4) I'm not sure – help?<br><br>If 1: Great, your daily preparation text message guide will begin tomorrow. You may read the instructions in full or watch an instructional video here: https://w2h.us/prepguide<br><br>If 2: Ok, we're sorry that this is not the program for you. Please follow the preparation instructions that were provided to you by your provider. Your messaging will now be turned off and this program will no longer be active. If you have received this message in error, reply with the word PREP to resume your preparation program. Thank you! (W2H Action: messaging automatically turns OFF)<br><br>If 3: Ok, we're sorry that this is not the program for you. You may read the instructions for your preparation process here: https://w2h.us/PEGPrep Your messaging will now be turned off and this program will no longer be active. If you have received this message in error, reply with the word PREP to resume your preparation program. Thank you! (W2H Action: messaging automatically turns OFF)<br><br>If 4: We will notify a staff member to help clarify which preparation process you should follow. Someone will reach out to you as soon as possible, but if you need to reach someone more quickly, please call {{@clinic_number}}. (W2H Action: In-basket msg sent to Coordinator)<br><br>If PREP: Your colonoscopy preparation program (using the Miralax/Gatorade preparation) will now resume. If you have questions or need assistance, reply with the word SUPPORT. (W2H Action: messaging automatically turns ON)

Day 7, 8am	It's officially prep week! We hope this program will help you through your prep! Please review this list of medications carefully: https://w2h.us/prepguide If you are on any of those listed, any blood thinners (like Coumadin, Warfarin, Eliquis, Xarelto, Pradaxa), or any other medication for diabetes, ask your prescribing doctor if you should adjust your medications before this procedure. Please do this as soon as possible. If you are taking an iron supplement, please stop this immediately until after your procedure. Other medications should be taken as instructed.
Day 6, 8am	Good morning, PARTICIPANT_FIRSTNAME! Don't forget to purchase your prep supplies from the local pharmacy or grocery store! Your prep includes:<br>- MiraLAX: 1 bottle, 238g<br>- Dulcolax: 4 pills, 5 mgs each<br>- Clear electrolyte drink: 64 ounces<br>Clear electrolyte drinks include Gatorade, PowerAde, Propel Fitness Water, or Pedialyte (yellow, green, or clear flavors ONLY). Please use the zero sugar versions if you are diabetic.
Day 5, 8am	Good morning, <name>! Let's take a look at your diet in preparation for your colonoscopy. Fiber is hard to purge from your system while doing a bowel prep. Starting today, please limit high fiber foods like fruits, veggies, nuts and seeds, and focus on low-fiber foods such as yogurt, eggs, white bread, rice, chicken, pork, and fish<br>It is also extremely beneficial to start drinking more water. The better hydrated you are, the more success you will have with your prep. Remember you can text "Support" at any time for additional information.
Day 3, 8am	Hi PARTICIPANT_FIRSTNAME! Remember to avoid high-fiber foods: fruits, vegetables, nuts, and seeds<br>Eat Low-Fiber foods: yogurt, egg, white bread, rice, chicken, pork, and fish. Do you have any questions we can answer for you today? Reply Y or N.<br>If N: Great, you're a pro! Text "Support" at any time if you change your mind.<br>If Y: Ok, in a few words, please tell us what you need additional assistance with and a staff member will get back to you within one business day. If you need to speak with someone more quickly, please call: {{@clinic_number}} for assistance. If this is
Day 2, 2pm	Starting tomorrow morning, you should only have clear liquids until your procedure is complete. Reminder: have all of your prep supplies on hand. For complete instructions, click here:<br>https://w2h.us/prepguide [attachment]:<br>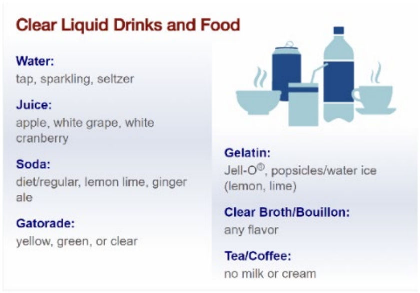
Day 2, 5pm	Please enjoy your dinner (with low fiber foods)! This will be your last meal with solid food before

	your colonoscopy. Remember, as soon as you wake up tomorrow, consume ONLY clear liquids until your procedure is complete.
Day 1, 8am	Good morning PARTICIPANT_FIRSTNAME! It's game time. You may only consume clear liquids from now until your procedure is complete. You can do this! More instructions to follow this afternoon when it's time to start your prep.
Day 1, 4pm	Time to get started.! Take the 4 Dulcolax pills now. Then, at 5 PM, mix the entire bottle of MiraLAX with the 64 oz of an electrolyte drink (Gatorade or Powerade, etc) and drink the first half of this mixture (32 oz). If you feel nauseous, you can always slow down to help tolerate it. Try drinking about 8 ounces every 15 minutes. We'll let you know when to drink the other half. You can do it!
Day 1, 8pm	Just to confirm, your arrival time is {{@arrival_time}} tomorrow. Great job, the first half of your prep is complete! Start drinking the other half of the MiraLAX solution 6 hours before your arrival (at {{@prep_time}}), and stop drinking all clear liquids 3 hours before at (at {{@stopliquids_time}}). If you have any questions overnight, please text Support. You're in the final stretch
Day 0, 5am or 3 hours before arrival time	You've come so far and the worst is over! Wear comfortable clothes today, and leave your valuables (like credit cards, cell phone, cash and jewelry) at home or with your ride. Please be SURE to bring your cell phone. Your appointment is at {{@clinic_address}}. Please arrive by {{@arrival_time}}.<br>When you arrive, please [parking and building instructions]. Your ride may come in with you. Congratulations! You did it
Support- help on-demand	A good prep is key to your procedure! We'll do our best to answer your question, please reply with the number (1-6) of the area you need help:<br>1: Appointment Info<br>2: Ride<br>3. Diet<br>4. Prep supplies and instructions<br>5. Prescriptions<br>6. Something else<br>After auto reply for any topic listed:<br>Does this answer your question? Reply Y or N<br>If Y: Great – we're happy we could help! You can text "Support" again at any time if you have any additional questions.<br>If N or Option 6: We're sorry our automated support couldn't help you with that. We will have someone reach out within the next business day. If you need to reach someone more quickly, please call the {{@clinic_name}} office directly at {{@clinic_number}}. (In-basket msg sent to Coordinator)

Supplement Figure 1. Pre and Post-Intervention Period Completion Rate for Control and Intervention

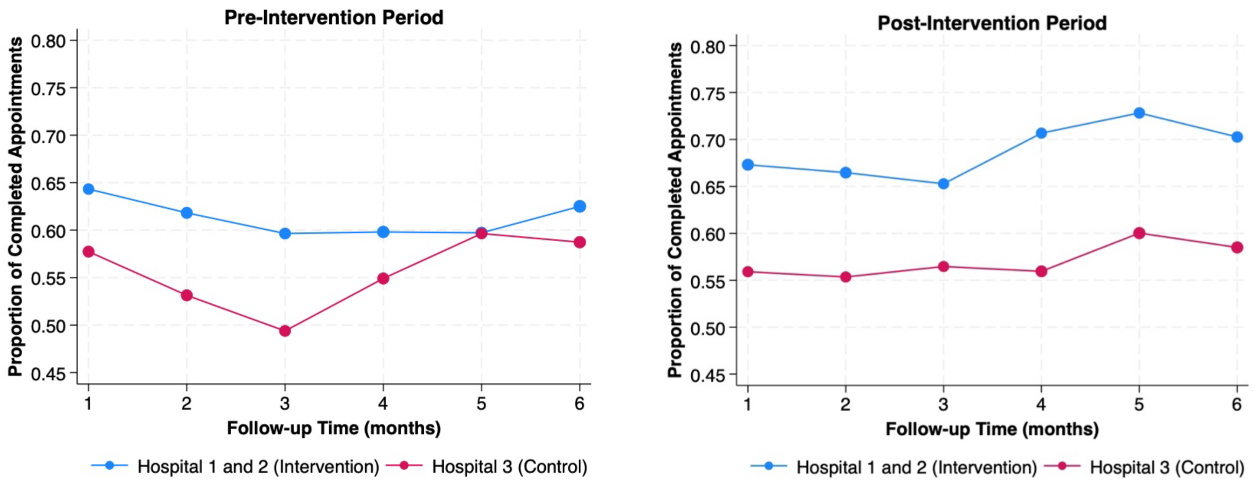

Supplement: Extended PDF [file mmc2.pdf]
